# Drought Resistance Loci in Recombinant Lines of Iranian *Oryza sativa* L. in Germination Stage

**DOI:** 10.3390/biotech10040026

**Published:** 2021-11-06

**Authors:** Morteza Noryan, Islam Majidi Hervan, Hossein Sabouri, Faroukh Darvish Kojouri, Andrea Mastinu

**Affiliations:** 1 Department of Plant Breeding, Science and Research Branch, Islamic Azad University, Tehran 1477893855, Iran; noryan1972@gmail.com (M.N.); majidi_e@yahoo.com (I.M.H.); farrokh_darvish@yahoo.com (F.D.K.); 2 Department of Plant Production, Collage of Agricultural Science and Natural Resources, Gonbad Kavous University, Gonbad Kavous 4971799151, Iran; 3 Department of Molecular and Translational Medicine, Division of Pharmacology, University of Brescia, 25123 Brescia, Italy

**Keywords:** germination, water shortage stress, *Oryza sativa*, QTL, SSR

## Abstract

In order to locate control genes related to *Oryza sativa* L. traits at the germination stage under normal conditions and at drought stress levels (−4.5 and −9.0 bar), we evaluated 120 F8 generation offspring from the cross between two cultivars Neda × Ahlemitarum in a factorial experiment in a completely randomized block design with three replications in 2013 in the botanical laboratory of Gonbad Kavous University. A linkage map was prepared using 90 Simple Sequence Repeats (SSR) markers and 28 Inter Simple Sequence Repeats (ISSR), and 6 iPBS and 9 IRAP markers (265 polymorphic alleles). The results of the analysis of variance showed that all of the evaluated traits had a significant difference at the probability level of 1%. Hence, it can be noted that the desired genetic diversity can be found between genotypes. The results of the stepwise regression analysis for the germination percentage as a dependent variable and other traits as independent variables in the studied treatments showed that under normal conditions, there was variable coleoptile length, but under drought stress of −4.5 and −9.0 bar, the variable plumule dry weight entered the model. In this study, the markers included in RM1-RM490 and ISSR2-3-RM133 of chromosomes 1 and 6 of *Oryza sativa* were identified as the main regulators of traits associated with *Oryza sativa* drought resistance. In particular, they present the quantitative trait loci (QTL) that control the first stages of germination of *Oryza sativa* in water stress conditions.

## 1. Introduction

Currently, water is rare in many parts of the world, and it is predicted that climate change will exacerbate this in the future. Water shortages are destroying huge volumes of agricultural products around the world, so they pose a serious threat to sustainable agriculture [1,2,3,4]. Plant adaptation to abiotic stress is divided into avoidance and tolerance [5,6,7,8,9,10,11]. Drought avoidance is associated with water uptake. These phenomena are related to root morphology and in particular, to the length of root [12,13]. 

Rice (*Oryza sativa* L.), which is the main food source for more than three billion people, accounting for 50–80% of their daily caloric intake, plays a major role in human nutrition [14,15,16]. Drought stress severely affects rice production. It has been claimed that drought has affected more than 23 million hectares of rain-fed rice in Asia [17]. With reducing agricultural water resources e around the world, the need to increase drought condition adaptation and to identify a variety of drought-resistant traits has become increasingly important [3,9,18,19,20].

Since water is considered to be one of the main requirements for seed germination, water stress caused by polyethylene glycol (PEG) greatly seed germination ability [21]. Seed germination and seedling growth are very important for the early establishment of plants under stress conditions [22,23]. The selection of genotypes for rapid and uniform germination under water stress can help to establish early seedling. Hence, an analysis of germination traits and their response to drought can be useful for selecting drought-tolerant *Oryza sativa* L. (*O. sativa*) genotypes. *O. sativa* is sensitive to drought stress and in response to drought stress, shows several morphological changes at different stages of growth [24]. However, *O. sativa* sensitivity to drought or water stress varies in terms of time, duration, intensity of drought stress, and variety and growth stage [25]. It has been reported that seed germination and seedling growth stage is one of the most important stages for water stress [26], results from studies have shown that drought stress reduces seed germination [27,28], seedling height [25], and the number of tillers in *O. sativa* [29]. 

Plenty of QTLs have been detected for the morphological traits involved in drought tolerance. This has helped to develop an understanding of the genetic structure that is responsible for drought-tolerance given that adaptation against drought stress. In particular, different genomic loci are responsible for controlling the drought-tolerance of *O. sativa* at different growth stages. For example, MacMillan and colleagues identified six QTLs for root-related traits in four different loci in *O. sativa*, most of which were QTLs for the same trait in four different loci [30]. In contrast, there are genomic loci that are involved in tolerating different stresses. For example, Wang and colleagues identified a QTL (qIR-4) that is associated with seed germination on *O. sativa* chromosome 4, which was associated with tolerance to salinity and drought stress [31]. Markers associated with these QTLs can be employed for marker-assisted selection to develop *O. sativa* cultivars that could be adapted to drought stress. Several major QTLs for drought-facilitated grain yield and its components have become present in different plants. For example, under the Generation Challenge Programmed in CIMMYT, Mexico, and the National Initiative on Climate Resilient Agriculture Project in India, efforts were made to insert QTLs for several drought-related traits into two elite Indian wheat cultivars (HD2733 and GW322) through marker-assisted backcrossing [32] (for details on QTLs, see [33,34,35,36]).

Given the greater sensitivity of the new *O. sativa* varieties to drought stress [19], it is necessary to select cultivars that are very resistant to drought through the use of molecular markers. Therefore, the aim of the present study is to identify and compare the QTLs of drought tolerance in the F8 population of *O. sativa* in the absence and presence of water stress.

## 2. Materials and Methods

### 2.1. Phenotypic Evaluations

In this study, the drought tolerance of the germination stage of an (recombinant inbred line) RIL *O. sativa* population including 120 F8 lines obtained from the cross of two cultivars Neda × Ahlemitarum was evaluated in a factorial experiment in a completely randomized design with three replications in the botanical laboratory of Gonbad Kavous University. Ahlamitarum and Neda cultivars are tolerant and sensitive to salinity, drought stress, and deficiencies of some mineral elements. These cultivars were selected to undergo different biotic and abiotic stresses during different breeding programs of the Iranian *O. sativa* germplasm at Gonbad Kavous University. 

**Figure 1:**
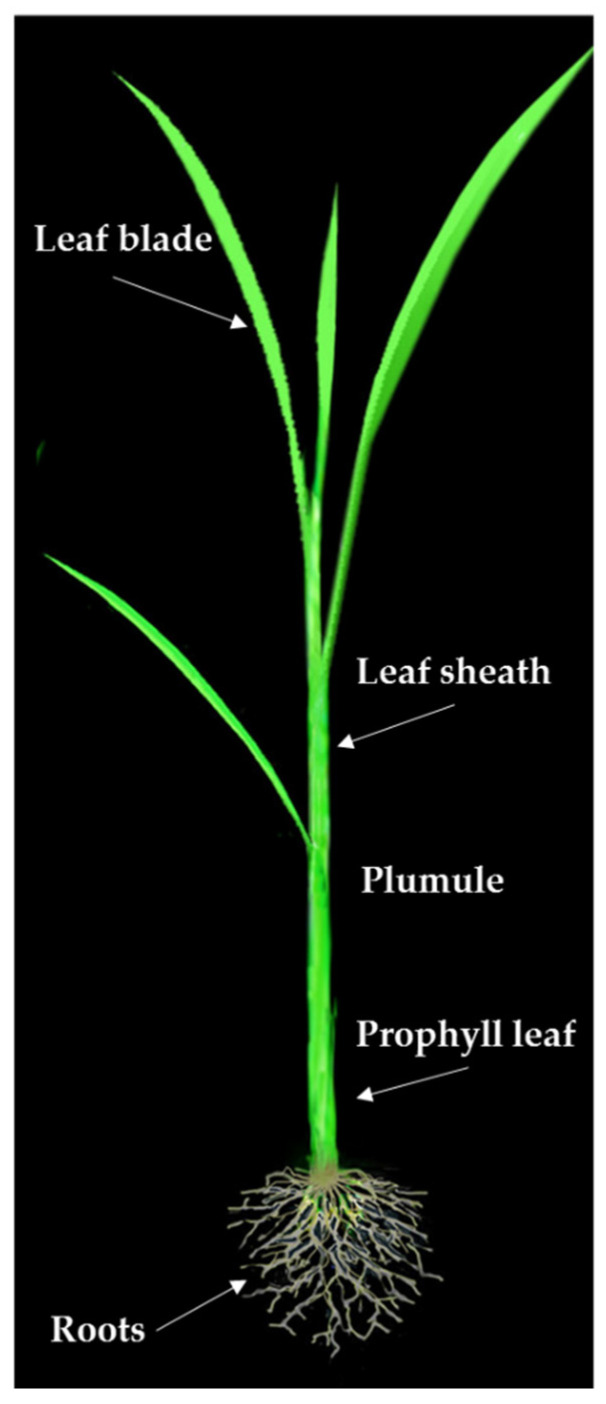
Morphology of a rice seedling.

The studied treatments included water and PEG 6000 solution at two levels of drought (−4.5 and −9.0 bar) and in a situation without drought stress (distilled water), respectively. A total of 100 healthy seeds per replication were selected from each genotype, and their surfaces were decontaminated with 2% sodium hypochlorite solution for 10 min and were then rinsed three times with distilled water. The seeds were then placed in sterile Petri dishes on sterile filter paper and were later placed in a germinator with a temperature of 25 °C and at 70% humidity and in dark conditions for one week. Dry treatments were applied for 14 days. After 14 days, 25 seedlings were randomly selected from each plate, and radicle, coleoptile, and plumule length and weight was measured (Figure 1). 

Germination percentage (PG) was based on the germination index and at least 2 mm root germination and was calculated according to Equation (1).

PG = Ni/N × 100(1)

where PG = percentage of germination, Ni = number of seeds germinated on the last day, and N = total number of seeds. Additionally, in order to calculate the rate of germination (RG), Equation (2) was used:

RG = X1/Y1+ (X2 − X1)/Y2 +…. + (X_n_ − X_n − 1_)/Yn (2)

Yn = number of days from the beginning of planting to day n, and X_n_ = number of seeds germinated on day n. 

The amount of polyethylene glycol used to create the necessary potential was obtained from Equation (3) [37]. PEG is a high-molecular-weight, non-ionic, and non-plasmolyzing compound that imitates drought stress in cultured cells similar to that observed in the cells of intact plants subjected to drought conditions [38]

φ = − (1.18 × 10^−2^) C − (1.18 × 10^−4^) C2 + (2.67 × 10^−4^) CT + (8.39 × 10^−7^) C2T (3)

where φ is osmotic potential, C is the concentration of polyethylene glycol 6000 in grams per liter, and T is the temperature in degrees Celsius.

### 2.2. Genotypic Evaluations

For genotypic evaluation, the DNA of the lines and parents of fresh leaves was analyzed [39]. The 285 SSR markers that were appropriately distributed on 12 chromosomes in the *O. sativa* were selected according to previous works [39,40,41]. These SSR primer pairs were examined for polymorphism between two parents, and polymorphic primers were used to amplify the DNA fragments of each plant from the RIL population. Given that both parents are *Indica* type for population development, the number of polymorphic markers was low. Since chromosome 1 in *O. sativa* has a more important role in controlling traits, in this study, more markers were used for this chromosome. In this study, 90 SSR markers and 28 ISSR, 6 iPBS, and 9 IRAP markers (265 polymorphic alleles) were used to identify the chromosomal position and to investigate the polymorphism of the studied lines. In order to perform a polymerase chain reaction, the iCycler thermos-cycler (BIORAD, Hercules, California, USA) was used. The thermal cycles for the SSR markers included an initial denaturation stage for 2.5 min at 95 °C, 35 cycles with denaturation at 95 °C for 1 min, primer binding for 30 s at their specific temperature, extension at the temperature was 72 °C for 30 s, and finally, the final propagation stage was for 5 min at 72 °C. Thermal cycles for the ISSR markers included an initial denaturation stage for 5 min at 95 °C, a denaturation stage at 95 °C for 1 min, 10 primer binding cycles at 42–54 °C for 1 min, expansion at 72 °C for 1 min, 25 primer binding cycles at their specific temperature for 45 s, and finally, a final amplification stage at 72 °C for 5 min. Polymerase chain reaction products were segregated using denaturation 6% polyacrylamide gel electrophoresis, and the bands were visible with a rapid silver staining method.

### 2.3. QTL Linkage and Analysis Map

A genetic map of the SSR, iPBS, IRAP, and ISSR markers was provided using the QTXB17 Map Manager Software (https://mapmanager.net/, (accessed on 2 November 2021)) [42]. The SSR and ISSR markers were used to provide a genetic map of the 120 F8 generation genotypes obtained from the Ahlemitarum and Neda cultivars. The mapping was completed with 120 lines. The expected ratio of 1:1 for the segregation of amplified markers in the evaluated lines was determined by χ^2^ test using Map Manager Software. In order to assign each of the markers to the relative chromosome, the linkage groups obtained here were compared to the previously reported genetic maps [39,40,41]. To convert the recombination ratios between markers to a map unit CentiMorgan (cM), KOSAMBI’S (1944) mapping function was used.

## 3. Results

The results of the analysis of variance (Table 1) under normal conditions and drought stress of −4.5 and −9.0 bar showed that a statistically significant difference was found between the studied lines at the 1% level for all of the evaluated traits, which indicates diversity between the studied lines of the measured traits. 

Forward stepwise regression was used to determine the relationship between germination percentage and other components of germination. For this purpose, the germination percentage trait was considered as a dependent variable, and other traits were considered as independent variables. For the trait percentage of germination under normal conditions, the variable coleoptile length was 0.039, under the drought conditions −4.5 bar, the plumule weight explained 0.327% of the total changes, and under the conditions of −9.0 bar, the plumule weight explained 0.272% of the total changes (Table 2).

**Figure 2:**
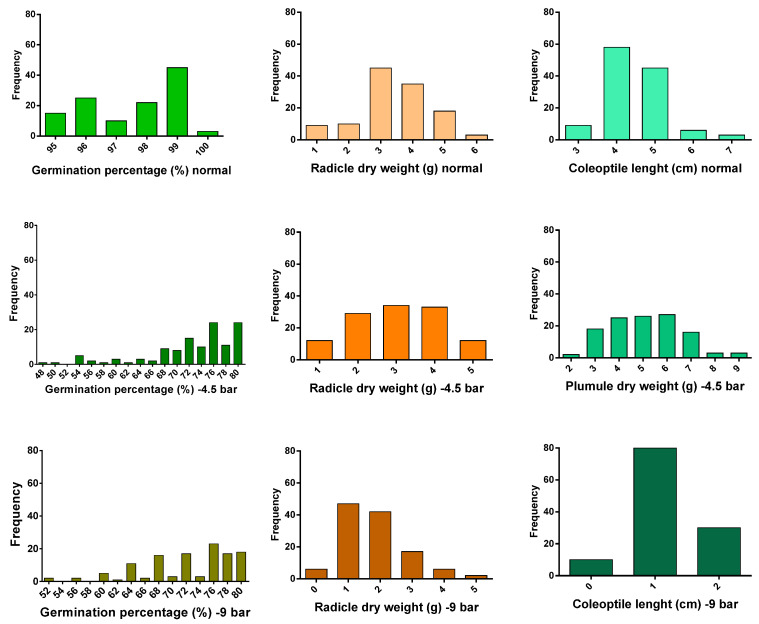
Phenotypic distribution of traits under normal conditions and drought stress levels (−4.5 and −9.0 bar) in 120 F8 families at the germination stage. X is the value of the traits, and Y is the frequency.

Figure 2 shows frequency distribution for the traits related to the germination stage under normal conditions and under drought stress levels of −4.5 and −9.0 bar. 

**Figure 3:**
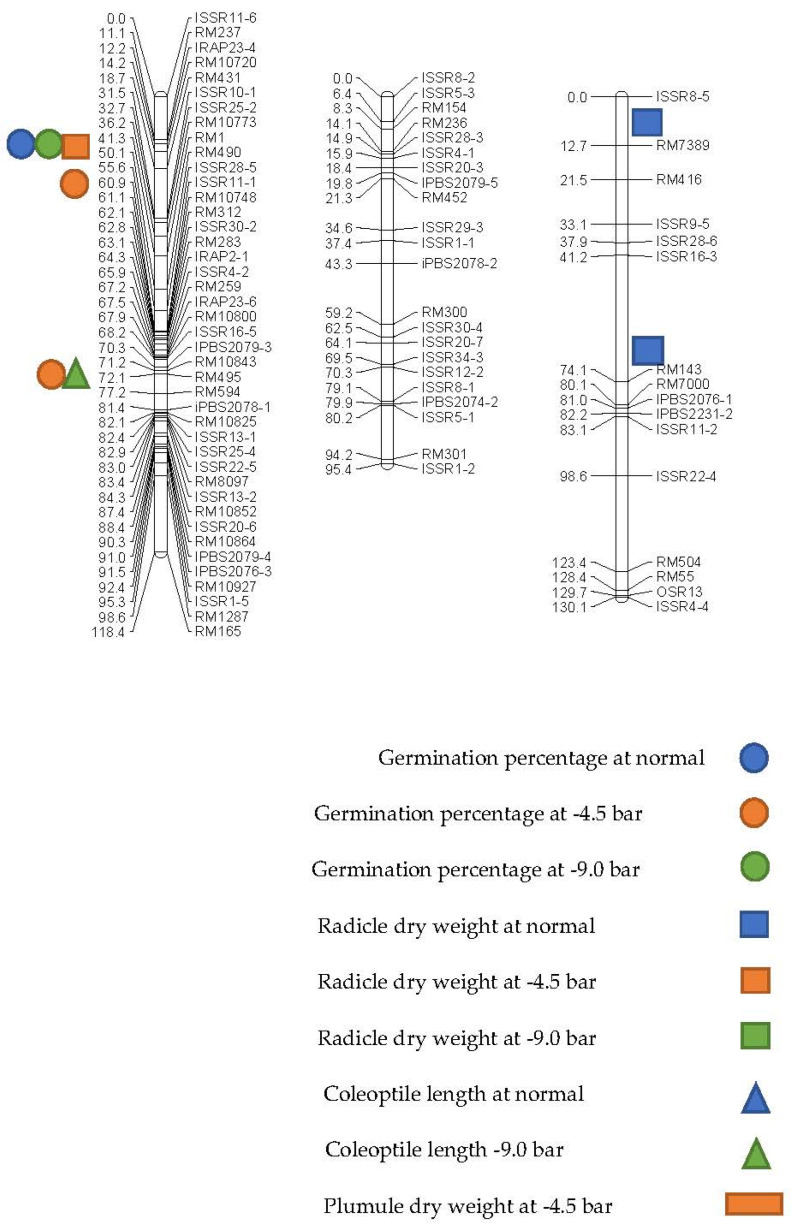

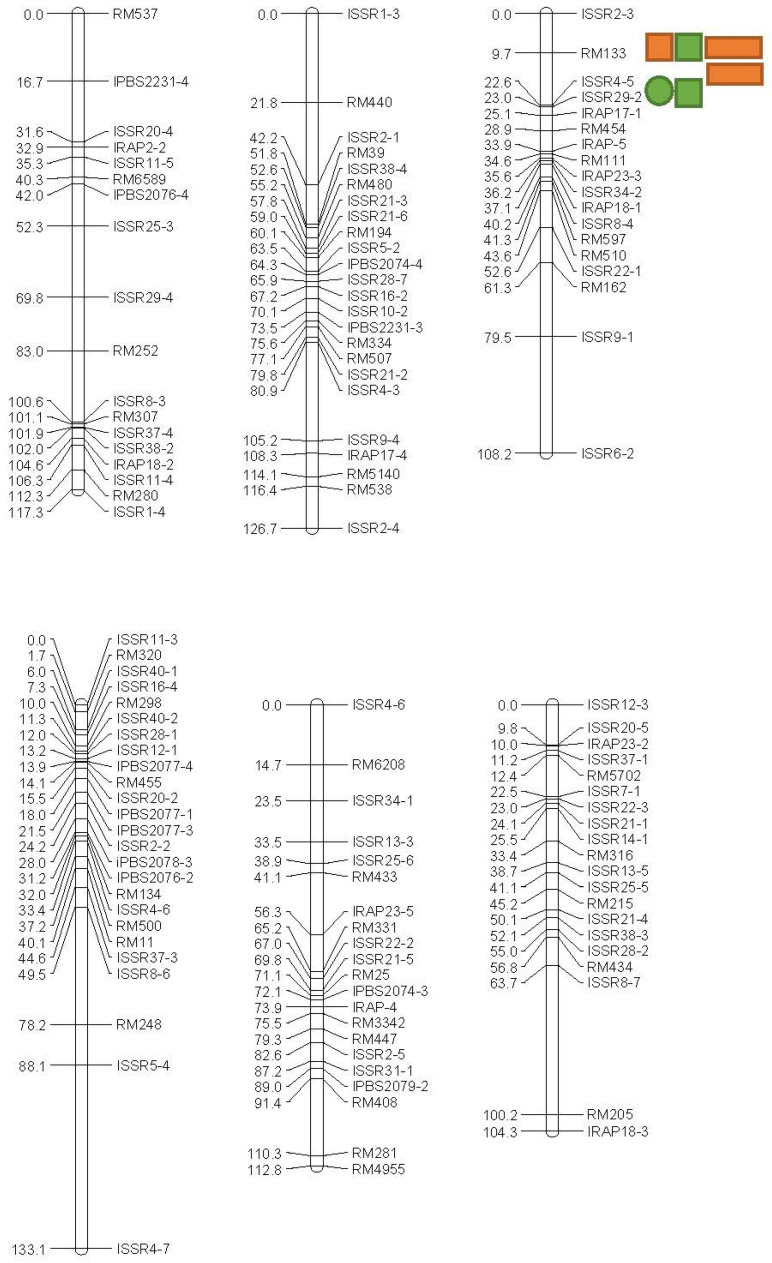

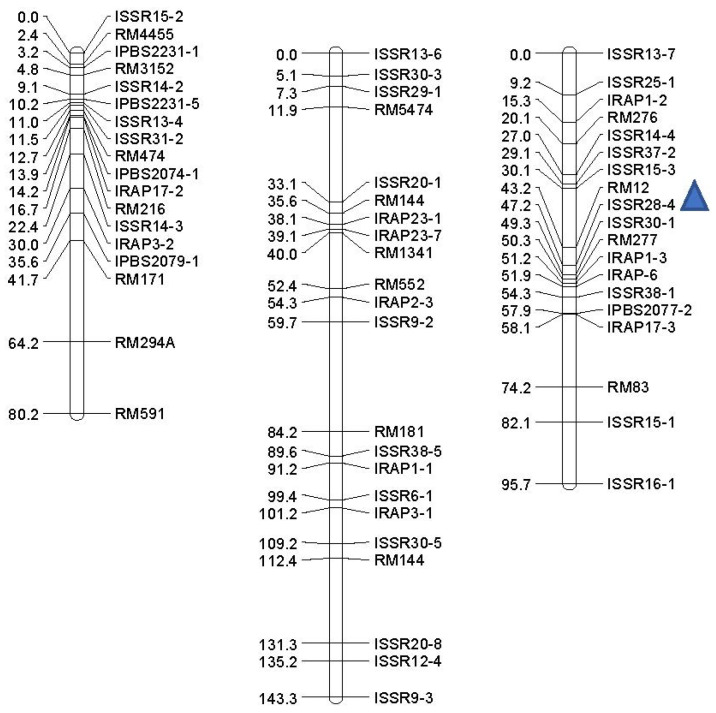
Location of controlling drought tolerance traits in the F8 population of *O. sativa* at the germination stage.

Under non-stress conditions, a total of four genomic loci were located for the evaluated traits at the germination stage, which controlled three traits. A control locus (qGP-1) was located for the germination percentage on chromosome 1 at the 60 cM position, which explained 15.5% of the phenotypic changes. The additive effect for this QTL was decreased, and Ahlemitarum’s parent alleles reduced this trait. In addition, two QTLs called qRDW-1 and qRDW-3 were identified on chromosomes 1 and 3 for the radicle dry weight. The coefficient of determination for the above two QTLs was 14 and 14.5%, respectively. This additive effect was also decreased due to the parent of Ahlemitarum reducing this trait. A QTL (qCOL-12) was identified on chromosome 12 at the RM12-ISSR28-4 marker interval for the coleoptile length trait, which explained 13.9% of the phenotypic changes. The additive effect was decreased and transferred from the parent of Ahlemitarum to the offspring (Table 3 and Figure 3). 

Under the drought stress conditions of −4.5 bar, seven QTL-containing loci were identified using the composite interval locating method for germination-related traits [5,28]. Two QTLs were identified for the germination percentage trait on chromosome 1. At the RM1-RM490 marking interval, the QTL (qGP-1a) was identified as having a large effect QTLT, with an LOD equal to 6.26 and a coefficient of determination of 21.3. For the radicle dry weight trait, two QTLs were located on chromosomes 1 and 6 at the genomic intervals of RM1-RM490 and ISSR2-3-RM133. The coefficients of determination for these two QTLs were 14.8 and 14.5, respectively (Table 3 and Figure 2). Additionally, three QTLs for plumule dry weight were identified on chromosomes 1 and 6, and six were identified at the genomic intervals of RM1-RM490, ISSR2-3-RM133, and RM133-RM4-5 and at positions 42, 8, and 18 cM, respectively. The coefficient of determination was high for each of the identified QTLs. However, QTL that as identified at the RM1-RM490 marking interval was as the largest effect QTL because it had the highest coefficient of determination (Table 3 and Figure 3). 

Under the drought stress conditions of −9.0 bar, six genomic loci controlling the evaluated traits at the germination stage were identified. Two QTLs on chromosomes 1 and 6 were involved in controlling the germination percentage trait, which together explained 34.6% of the phenotypic changes. The QTL identified at the marker interval RM1-RM490 was a large effect QTL at this stress level due to a higher coefficient of determination than other QTLs. The additive effect for these two QTLs was positive, and the parental alleles of the Neda cultivar increased this trait (Table 3 and Figure 3). For the coleoptile length trait, a QTL was located on chromosome 1 at the RM495-RM594 genomic intervals. This QTL was identified as a large effect QTL because it had the highest coefficient of determination, which was equal to 18% (Table 3 and Figure 3).

A comparison of the identified QTLs shows chromosomes 1 and 6 as common chromosomes and genomic locus RM1-RM490 as a common locus under stress conditions for when the germination percentage trait was between 17.8 to 21.3%.

## 4. Discussion

To explain the complexity of the drought stress response and the investigation of the quantitative genes associated with these responses, we used a genomic technology called QTL mapping. In this study, we recorded the germination attributes in the mapping population and the identified QTLs for drought tolerance in *O. sativa*. Additionally, the RIL population showed transgressive segregation for such traits. For most of the evaluated traits, having values that were higher and lower values than those of the parents indicated transgressive segregation for the studied traits (Figure 2). The presence of transgressive segregation increases the likelihood of identifying QTLs for traits and indicates that both parents contain desired and undesired alleles in different traits. In a study on *O. sativa* that observed transgressive segregation for all of the traits studied Tian and colleagues stated that this phenomenon could be due to the recombination of small QTLs and interactions between genotypes and the environment [43]. 

The difference between the lines was significant, and this prompted us to track the QTLs controlling germination-related traits. Woźnicka and colleagues showed that a significant difference was found in the analysis of variance between most of the studied traits at the 1% level [44].

The linkage map of the 90 SSR markers and the 28 ISSR, 6 iPBS and 9 IRAP markers (265 polymorphic alleles) on 120 individuals in the F8 population divided the markers into 12 linkage groups belonging to 12 *O. sativa* chromosomes, with a map length of 1365.3 cm and an interval of 5.17 cm between two adjacent markers. According to Lander and Botstein [45], if the average distance between two markers is less than 20 cM, the linkage map will be used to detect QTLs. The maximum coupling length was on chromosome 11, with 143.3 centiMorgan (cM), and the minimum coupling length was 80.2 cm on Chromosome 10. Other researchers [28,46] have also drawn different genetic maps in different populations of Iranian *O. sativa* where the differences can be attributed to the population type, the genetic background of the populations used as well as the type and number of markers used in various studies.

The clustering of QTLs for some traits are often mapped in the same chromosomal regions [5,6,47,48,49]. This trend was also observed in this study. For example, qGP-1a, qRDW-1, qPDW-1 (at −4.5 bar) and qGP-1, and qRDW-1 (at −9.0 bar) were located on chromosome 1 at the same map locations. In these cases, the directions of the correlations were consistent with that of the effects of the QTLs on the traits. These results support the fact that the trait correlation may be attributed to the effect of pleiotropy or to the very close linkages of these genes.

Hu and colleagues used a recombinant inbred population from the cross between CO39 (*Indica*) and Moroberekan (*Japonica*) cultivars to investigate fresh and dry weight control QTLs of radicle under drought stress in *O. sativa* [50]. They identified a QTL on chromosome 1 with an LOD equal to 4.7 and with 21% of the total phenotypic variance, which was consistent with the present results. Under normal conditions, Najeeb and colleagues also identified seven QTLs for coleoptile length on chromosomes 1 (two cases), 2 (two cases), 7, 11, and 12, which explained 12.16% of phenotypic changes in the trait [51]. 

In another study, Sabouri and colleagues determined two QTLs in relation to plumule dry weight on chromosomes 1 and 3. The QTLs had additive effects of −1.01 and −0.04, respectively, and a phenotypic explanation of 22.44 and 22.97 [41]. 

Using a recombinant population of crosses between Indica (IR24) and Japonica (Asominoria) cultivars to investigate QTLs associated with low-temperature germination using the RFLP marker, Jain and colleagues identified 15 QTLs that were related to germination percentage on chromosomes 2, 3, 7, 10, 11, and 12 [52]. In addition, Wang and colleagues identified seven QTLs using the RIL population (F2: 9) that was located on chromosomes 6, 7, and 10, which are similar to the QTLs identified in another study [31].

Liu and colleagues identified two QTLs for this trait on chromosomes 4 and 11 using the composite interval locating method for F3 generation *O. sativa* population resulting from the cross of the Gharib and Sepid Roud cultivars [53]. Three QTLs were identified for radicle dry weight on chromosomes 1 and 6 at genomic intervals RM1-RM490, ISSR2-3-RM133, and RM133-ISSR4-5, with coefficients of determination equal to 35.1, 27.3, and 26.1%, respectively. The additive effect was true for these three QTLs, and the Neda parent alleles increased this trait. Additionally, a QTL located at the RM1-RM490 marker interval was identified as a large effect QTL due to the explanation of the highest percentage of phenotypic changes in this trait. The phenotypic locus and variance described by qGP-6 are similar to the QTL identified by Teng and colleagues [54]. The reasons for the inconsistency between the results of the present study and the results of some researchers are the number and type of markers studied, type of population, number of individuals used in the population, the parents used, and environmental conditions. 

A great deal of research has considered root morphology as having an effect on drought tolerance at different growth stages [46,55,56]. Some QTLs for root length are located on chromosomes 1 (linked to RM243 and RM23) and 9 (linked to RM257 and RM258) at the seedling stage [57]. QTLs related to radicle dry weight and germination percentage can also be considered as stable QTLs because they can be found under three conditions. Chromosomes 1 and 6 and the genomic loci RM1-RM490 and ISSR2-3-RM133 were also identified as common chromosomes and loci for the radicle dry weight trait because the QTLs related to this trait are commonly on these two chromosomes and loci under three conditions. Srividhya and colleagues found five stable QTLs among twenty-four identified cases for traits related to normal conditions and drought stress induced by PEG at the seedling stage in *O. sativa* [58]. In addition, Kamoshita and colleagues only found two stable QTLs out of the thirty-one identified cases for seven traits related to root morphology in two experiments with different planting dates [59]. 

Given the delicate balance between plants and arid ecosystems [4,7,10,11,60], in this research, QTL mapping using SSR and ISSR markers identified 17 QTL for germination parameters. These QTLs could transfer important genes for drought adaptability. These QTL could be applied for fine mapping, gene discovery, and marker-assisted selection in *O. sativa* breeding. The study proposes the combination of molecular markers with appropriate phenotyping as a suitable approach for complex traits such drought to select plants with a higher germination advantage under drought stress. 

## 5. Conclusions

The problem of drought associated with climate change is the main cause of reduced productivity of crops of agronomic value in developing countries. In particular, Iran belongs to a geographical area afflicted by a drought climate. Furthermore, rice is one of the most important foods in Asia and the world that requires considerable amounts of water to develop. For all these reasons, there is a need to identify genomic, proteomic and metabolomic traits associated with drought resistance. In this manuscript, Iranian cultivars of *Oryza sativa* resistant to water stress during the germination and development stages were studied. In particular, it has been shown that some QTL regions of chromosome 1 and 6 of *Oryza sativa* are involved in resistance to drought. In the future, proteomic and metabolomic approaches will have to be applied to fortify agriculture and make it more resistant to significant climate change.

## Figures and Tables

**Table 1:** Analysis of variance of studied traits at germination stage under normal, −4.5 bar, and −9.0 bar conditions.

Source of Variation	Freedom Degree	Means of Square			
		Germination Percentage (%)	Coleoptile Length (cm)	Radicle Dry Weight (g)	Plumule Dry Weight (g)
Normal					
Genotype	119	6.860 **	1.690 **	36.919 **	22.422 **
Error	240	2.394	0.244	0.299	0.249
Coefficient of variation		1.586	11.306	5.377	5.782
−4.5 bar stress					
Genotype	119	262.698 **	0.362 **	6.517 **	5.347 **
Error	240	45.111	0.198	1.418	0.692
Coefficient of variation		9.403	29.925	23.350	22.567
−9.0 bar stress					
Genotype	119	131.387 **	0.368 **	12.510 **	6.444 **
Error	240	24.844	0.012	0.120	0.097
Coefficient of variation		6.969	8.488	10.402	10.285

**: significant at the probability level of 1%.

**Table 2:** Results of stepwise regression for germination percentage as a dependent variable and other traits as independent variables in germination experiments under normal conditions and drought stress levels (−4.5 and −9.0 bar).

Stress	Dependent Variable	Variable Entered the Model	B	Beta	SE	F	R^2^
No stress	percentage of germination	coleoptile length	99.290	−0.198	0.182	4.792	0.039
−4.5 bar	percentage of germination	plumule weight	4.009	0.572	0.529	57.349	0.327
−9.0 bar	percentage of germination	plumule weight	2.356	0.522	0.354	44.189	0.272

B: regression coefficient; Beta: standard regression coefficient; SE: standard error; F: F statistic, and R^2^: coefficient determination.

**Table 3:** Control QTLs for the studied traits at the germination stage under normal conditions.

Trait	QTL	Markers Interval a	Chr.	Position (cM) b	LOD	Additive Effect	(R^2^) c (%)	DPE d
Non-stress								
GP (%)	qGP-1	ISSR28-5-ISSR11-1	1	60	2.34	−3.476	15.5	Ahi
RDW (gr)	qRDW-1	ISSR16-3-RM143	3	68	2.47	−1.274	14	Ahi
	qRDW-3	ISSR8-5-RM7389	3	6	2.274	−1.65	14.5	Ahi
COL (cm)	qCOL-12	RM12-ISSR28-4	12	46	2.193	−0.292	13.9	Ahi
−4.5 bar								
GP (%)	qGP-1a	RM1-RM490	1	42	6.257	4.78	21.3	Ned
	qGP-1b	RM10843-RM495	1	72	5.407	4.664	18.7	Ned
RDW (gr)	qRDW-6	ISSR2-3-RM133	6	8	4.161	0.734	14.8	Ned
	qRDW-1	RM1-RM490	1	42	4.085	0.621	14.5	Ned
PDW (gr)	qPDW-1	RM1-RM490	1	42	13.426	0.937	40.3	Ned
	qPDW-6a	ISSR2-3-RM133	6	8	8.68	0.922	2.73	Ned
	qPDW-6b	RM133-ISSR4-5	6	18	8.579	2.228	28.1	Ned
−9.0 bar								
GP (%)	qGP-1	RM1-RM490	1	42	5.105	3.086	17.8	Ned
	qGP-6	RM133-ISSR4-5	6	10	4.803	2.993	16.8	Ned
COL (cm)	qCOL-1	RM495-RM594	1	76	5.174	0.177	18	Ned
RDW (gr)	qRDW-1	RM1-RM490	1	42	11.276	1.338	35.1	Ned
	qRDW-6a	ISSR2-3-RM133	6	8	8.32	1.385	27.3	Ned
	qRDW-6b	RM133-ISSR4-5	6	22	8.899	20.883	26.1	Ned

GP: germination percentage; RDW: root dry weight; PDW: plumule dry weight; COL: coleoptile length; Chr.: chromosome. a: Underlined markers are closer to QTL; b: QTL position from the nearest flanking marker (cm); c: phenotypic variance explained by each QTL. d: DPE, direction of phenotypic effect; Ahi and Ned indicate Ahlemitarum and Neda, respectively. LOD: logarithm of the odds.

## Data Availability

The data presented in this study are available upon request from the corresponding author.
